# *TBC1D24* emerges as an important contributor to progressive postlingual dominant hearing loss

**DOI:** 10.1038/s41598-021-89645-y

**Published:** 2021-05-13

**Authors:** Dominika Oziębło, Marcin L. Leja, Michal Lazniewski, Anna Sarosiak, Grażyna Tacikowska, Krzysztof Kochanek, Dariusz Plewczynski, Henryk Skarżyński, Monika Ołdak

**Affiliations:** 1 Department of Genetics, Institute of Physiology and Pathology of Hearing, M. Mochnackiego 10, 02-042 Warsaw, Poland; 2 Postgraduate School of Molecular Medicine, Medical University of Warsaw, Warsaw, Poland; 3 Laboratory of Functional and Structural Genomics, Centre of New Technologies, University of Warsaw, Warsaw, Poland; 4 Centre for Advanced Materials and Technologies, Warsaw University of Technology, Warsaw, Poland; 5 Department of Otoneurology, Institute of Physiology and Pathology of Hearing, Warsaw/Kajetany, Poland; 6 Department of Experimental Audiology, Institute of Physiology and Pathology of Hearing, Warsaw/Kajetany, Poland; 7 Laboratory of Bioinformatics and Computational Genomics, Faculty of Mathematics and Information Science, Warsaw University of Technology, Warsaw, Poland; 8 Oto-Rhino-Laryngology Surgery Clinic, Institute of Physiology and Pathology of Hearing, Warsaw/Kajetany, Poland

**Keywords:** Disease genetics, Genetic testing, Clinical genetics, Medical genetics, Mutation, Sequencing, Clinical genetics

## Abstract

Several *TBC1D24* variants are causally involved in the development of profound, prelingual hearing loss (HL) and different epilepsy syndromes inherited in an autosomal recessive manner. Only two *TBC1D24* pathogenic variants have been linked with postlingual progressive autosomal dominant HL (ADHL). To determine the role of *TBC1D24* in the development of ADHL and to characterize the *TBC1D24*-related ADHL, clinical exome sequencing or targeted multigene (n = 237) panel were performed for probands (n = 102) from multigenerational ADHL families. In four families, *TBC1D24*-related HL was found based on the identification of three novel, likely pathogenic (c.553G>A, p.Asp185Asn; c.1460A>T, p. His487Leu or c.1461C>G, p.His487Gln) and one known (c.533C>T, p.Ser178Leu) *TBC1D24* variant. Functional consequences of these variants were characterized by analyzing the proposed homology models of the human TBC1D24 protein. Variants not only in the TBC (p.Ser178Leu, p.Asp185Asn) but also in the TLDc domain (p.His487Gln, p.His487Leu) are involved in ADHL development, the latter two mutations probably affecting interactions between the domains. Clinically, progressive HL involving mainly mid and high frequencies was observed in the patients (n = 29). The progression of HL was calculated by constructing age-related typical audiograms. *TBC1D24*-related ADHL originates from the cochlear component of the auditory system, becomes apparent usually in the second decade of life and accounts for approximately 4% of ADHL cases. Given the high genetic heterogeneity of ADHL, *TBC1D24* emerges as an important contributor to this type of HL.

## Introduction

Increasing use of high throughput DNA sequencing methods has significantly improved the detection rate of genetic alterations causative of hearing loss (HL). It has resulted in discovering novel HL variants and genes and assigning new inheritance patterns to known HL genes^1,2^. Several HL genes are causally involved in the development of both autosomal recessive and dominant forms of hereditary HL. Another level of complexity is provided by the diversity of clinical presentation. Some HL genes may lead to isolated HL but also syndromes that do not necessarily include HL as one of their phenotypic features^3^. This phenomenon is well-exemplified by the *TBC1D24* gene.

Recessive variants detected in *TBC1D24* may cause a spectrum of phenotypes, beginning with a mild form of familial infantile myoclonic epilepsy (FIME; OMIM #605021) and encompassing early-infantile epileptic encephalopathy 16 (EIEE16; OMIM #615338) and progressive myoclonic epilepsy (PME^4^) that represent a combination of epilepsy with other variable features and ending with DOORS syndrome (deafness, onychodystrophy, osteodystrophy, mental retardation and seizures; OMIM #220500), a syndromic form of HL^5^. Alterations in *TBC1D24* were also found in patients with isolated HL inherited in autosomal recessive (DFNB86; OMIM #614617) or autosomal dominant manner (DFNA65; OMIM #616044). While the involvement of *TBC1D24* in the development of a recessive form of HL is documented, with ten pathogenic variants identified so far^6–10^, only two *TBC1D24* pathogenic variants have been reported in the context of autosomal dominant HL (ADHL)^1,11,12^.

More than 40 different TBC proteins (TBC domain-containing proteins) are present in humans. TBC1D24 (and its homolog from *D. melanogaster*—Skywalker) contains a unique combination of TBC and TLDc domains. The TBC domain is considered to serve as GTPase-activating protein (GAP) that promotes GTP conversion to GDP in Rab proteins, which inactivates them. The Rab proteins are a crucial component of vesicular trafficking and are involved in vesicle formation, cargo transport along the cytoskeleton, and membrane fusion^13^. In 2006, a dual-finger mechanism of the TBC-Rab activity was proposed by Pan et al.^14^. According to this mechanism, both the TBC domain and Rab supply residues crucial for GTP hydrolysis.

Interestingly, a different mechanism was postulated for the TBC domain of Skywalker, as the region that supposedly interacts with Rab, is substantially different from that observed for other TBC proteins^15^. This alternative hypothesis explains the TBC1D24 impact on vesicular traffic by the ability of its TBC domain to interact directly with the membrane. This hypothesis originates from the observation of the positively charged region unique for the TBC1D24 orthologs, constituting residues that are conserved among this family and are not observed in other TBC proteins. The binding is presumed to occur through direct interactions with phosphoinositides in the lipid bilayer as the TBC domain from Skywalker does not bind to membranes devoid of these lipids. The estimated K_d_ (dissociation constant) for IP_3_ binding is around 0.019 mM, similar to other phosphoinositide-binding proteins. Moreover, changes of the positively charged interface (e.g., Arg79Cys, Arg281Cys) significantly weaken the interactions with IP_3_ (K_d_ rises to 0.32 and 0.18 mM, respectively) and produce several phenotypes that can be linked to impaired vesicle transport, such as seizures. The role of TLDc, the second TBC1D24 domain is more obscure. However, oxidative stress sensing or resistance has been demonstrated in cell cultures^16^.

For this study we carefully selected 102 HL families with pedigrees showing an autosomal dominant mode of inheritance. We found that in four of them, HL developed due to a *TBC1D24* pathogenic variant. For three of the families, the identified variants were novel, not previously associated with hearing impairment. Stimulated by the relatively high frequency of *TBC1D24* variants in the ADHL cohort, we focused on molecular aspects of detected *TBC1D24* variants and clinical features of the *TBC1D24*-related HL. In this paper, we provide extensive characteristics of this type of HL.

## Materials and methods

### Ethics approval

All tested subjects gave informed consent for participation in the study following the tenets of the Declaration of Helsinki. The study was approved by the ethics committee at the Institute of Physiology and Pathology of Hearing (KB.IFPS.25/2017).

### Study subjects

**Figure 1:**
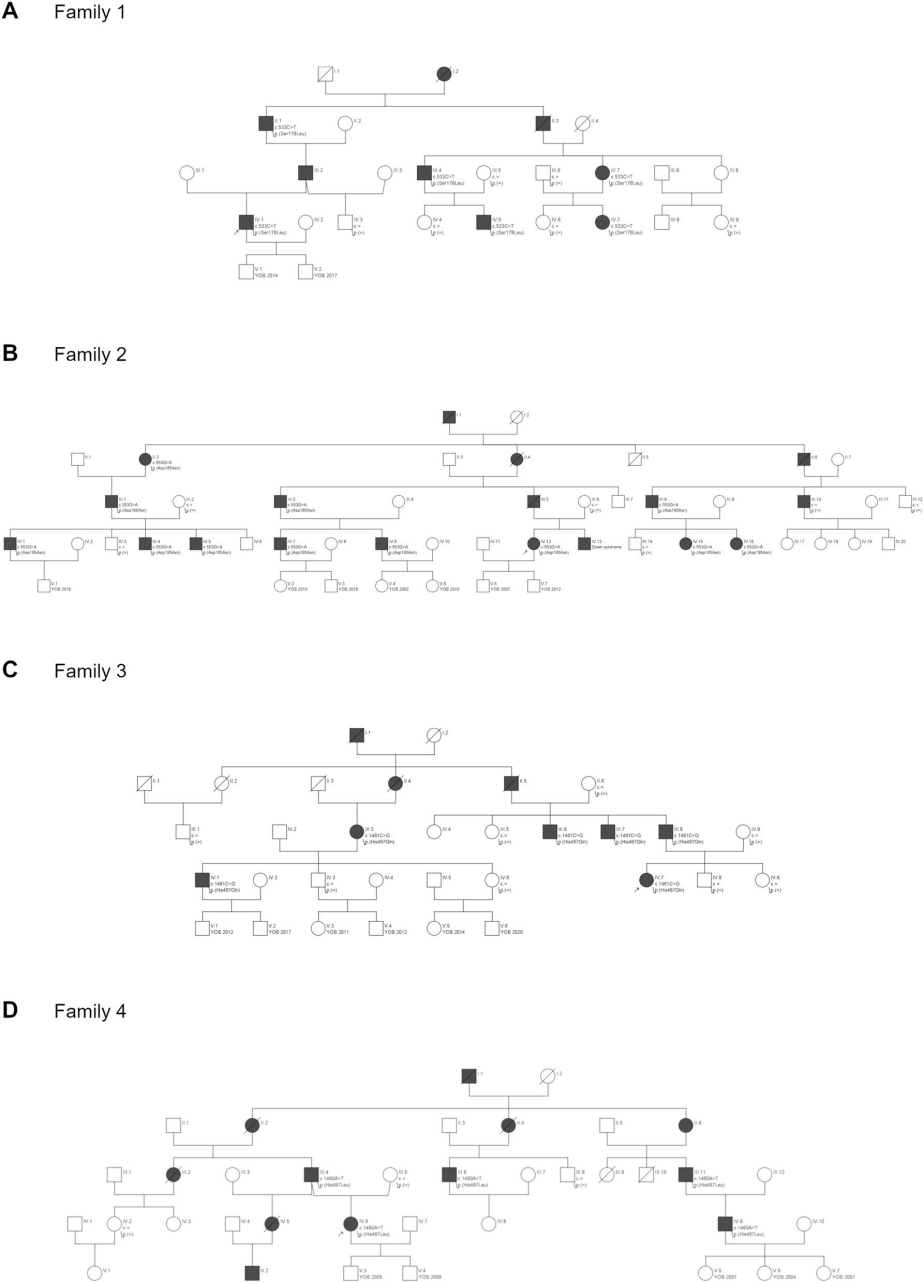
Four families with *TBC1D24* pathogenic variants. Black symbols indicate individuals affected by HL; open symbols indicate unaffected individuals; diagonal line denotes the deceased individuals. *YOB* year of birth. In Family 1 (**A**) *TBC1D24* c.533C>T (p.Ser178Leu), in Family 2 (**B**) *TBC1D24* c.553G>A (p.Asp185Asn), in Family 3 (**C)** *TBC1D24* c.1461C>G (p.His487Gln) and in Family 4 (**D**) *TBC1D24* c.1460A>T (p.His487Leu) were identified as causative for ADHL.

A total of 102 multigenerational families with HL occurring in at least three generations were selected for the study. In all recruited families, HL was transmitted from at least one male family member to offspring, which strongly indicated an autosomal dominant mode of HL inheritance. From this group, four families with *TBC1D24* pathogenic variants (n = 29 HL patients and n = 22 unaffected individuals) were selected for further evaluation (Fig. 1A–D).

### Audiometry and hearing threshold data analysis

Pure-tone audiometry (PTA) was performed in individuals from the tested families. Hearing thresholds were measured with the AC40 clinical audiometer (Interacoustics, Middelfart, Denmark) for frequencies 125–8000 Hz with 10/5 dB descending-ascending threshold estimation procedure^17^. The degree of HL was described as mild (21–40 dB), moderate (41–70 dB), severe (71–90 dB) or profound (> 90 dB). All available previous PTA results were also analyzed. Symmetry of hearing thresholds was validated, and mean binaural air conduction thresholds (dB hearing level, dBHL) were calculated. Patient III.10 from Family 2 has been excluded from further comparisons because his audiograms revealed steeply-sloping high-frequency HL different from all his family members. His HL most likely developed due to the acoustic trauma experienced in adolescence; none of his children had HL (Supplementary Fig. S1). Binaural hearing threshold data from families with *TBC1D24* p.Ser178Leu and p.Asn307His pathogenic variants reported in previous studies were also collected^1,11,12^.

Based on the obtained PTA data (n = 81 binaural hearing thresholds) from individuals with different *TBC1D24* pathogenic variants, the age-related typical audiograms (ARTA) were constructed as described previously^18^. A cross-sectional linear regression analysis of threshold on age was performed for every hearing frequency, and then characteristic hearing thresholds were predicted for fixed ages (10–80 years). The HL progression was determined and expressed as the annual threshold deterioration (ATD; dB per year). The progression was considered significant if the regression coefficient (slope) was significantly different from 0 at p < 0.05.

The ARTA were also constructed separately for *TBC1D24* p.Ser178Leu (Family 1 together with the previously published data for this variant^1,11^), as well as for p.Asp185Asn (Family 2) and p.His487Leu (Family 4). There were no longitudinal PTA data for separate ARTA calculations for the remaining *TBC1D24* p.His487Gln variant (Family 3). To evaluate differences between characteristic HL patterns observed in patients with different *TBC1D24* variants, threshold features arrays were calculated^19^. The data were plotted and compared using the chi-square goodness of fit test with significance level p < 0.05.

### Additional audiological and neurotological evaluation

For all probands (IV.1 Family 1; IV.12 Family 2; IV.7 Family 3 and IV.6 Family 4) additional audiological and neurotological examinations were performed. Assessment of auditory function comprised impedance audiometry, transient evoked otoacoustic emissions (TEOAE) and auditory brainstem responses (ABRs). Acoustic impedance measurements were performed with the Zodiac 901 instrument (Madsen Electronics, Copenhagen, Denmark). Stapedius reflex was analyzed for the frequencies 500, 1000, 2000 and 4000 Hz in the ipsilateral and contralateral modes^20^. TEOAE were evoked by standard nonlinear click stimulus with an intensity of 80 ± 5 dB peSPL and recorded using the ILO-292 system (Otodynamics Ltd, Hatfield, United Kingdom).

ABRs were recorded using the Integrity V500 system (Vivosonic Inc., Toronto, Canada). The stimulus was 0.1 ms click with alternating polarity presented with the 90 dB normal hearing level (nHL) intensity at repetition rates of 11/s and 37/s. The amplifier bandwidth was 30–1500 Hz and analysis time 11 ms. The number of sweeps required for an averaged response was 1024.

The objective vestibular function was assessed based on the cervical and ocular evoked myogenic potentials (cVEMP, oVEMP) recorded following stimulation with a 500 Hz tone burst (2 ms rise/fall time, 2 ms plateau) presented through 3 M E-A-RTONE insert earphones at an intensity of 97 dB nHL and a presentation rate of 5.1/s. VEMP measurements were performed using the Interacoustics Eclipse module system (EclipsVemp, Interacoustics, Assens, Denmark). oVEMP responses were considered present if they were stronger than noise, and the N1 and P1 latencies replicated exactly across multiple collections. Responses were required to be larger than 1.5 mV to be considered present. oVEMP interaural amplitude was considered significantly asymmetric and abnormal if there was over 33% side-to-side difference^21^. cVEMP interaural amplitude was considered significantly asymmetric and abnormal, if there was over 36% side-to-side difference^22^. To evaluate inner ear morphology and vestibulocochlear nerves anatomy, the temporal bone computed tomography was performed on a 64-slice CT scanner (Siemens CT Definition AS, Germany).

### Multigene panels and Sanger sequencing

Genomic DNA was isolated from whole blood samples and buccal swabs of available family members. In probands from Families 1 (IV.1), 3 (IV.7) and 4 (IV.6) a custom multigene panel containing 237 HL genes was performed (SeqCap EZ Choice, Roche, Basel, Switzerland) and the DNA libraries were run on MiSeq using 2 × 75 bp paired-end reads^23^. In the index patient from Family 2 (IV.12) clinical exome sequencing (TruSightOne, Illumina, Cambridge, UK) was performed according to the manufacturer’s protocol. The sample was run on MiSeq using 2 × 150 bp paired-end reads. Bioinformatics analysis was performed as described previously^24^. Selected reads and candidate variants were verified with the Integrative Genomics Viewer (IGV)^25^.

The analysis pipeline included variant population frequencies from different population databases, i.e., the UK10K project (https://www.uk10k.org/), the NHLBI GO Exome Sequencing Project (ESP) (https://esp.gs.washington.edu/drupal/) and the Genome Aggregation Database (gnomAD) (http://gnomad.broadinstitute.org) (all accessed in 03/2021). Pathogenicity predictions for non-synonymous variants were performed using REVEL^26^, CADD^27^, LRT^28^, PolyPhen-2^29^, SIFT^30^ and MutationTaster2^31^ computational algorithms. The potential effect of detected variants on *TBC1D24* RNA splicing was assessed using SpliceSite Finder, MaxEntScan, NNSPLICE, GeneSplicer algorithms integrated with Alamut Visual Software v2.15 (Interactive Biosoftware, Rouen, Paris). The pathogenic potential of identified variants was evaluated according to standards and guidelines for interpreting sequence variants^32,33^.

The presence of the candidate pathogenic variants was confirmed by Sanger sequencing and reported based on the *TBC1D24* NM_001199107.1 and NP_001186036.1 reference sequences. The primer pairs 5’-GTTCCCCGACATCTCCTTCTG and 5’-TTCTGCTTCAGGGCTTTCTCATT as well as 5’-GATGAAACGGGTTGTGGCTCT and 5’-CAGACCGTTGACCCTCCATAG were used for amplification of *TBC1D24* exon 2 and 7, respectively. PCR products were labeled with BigDye Termination cycle sequencing kit v3.1 (Applied Biosystems, Foster City, CA, USA) and sequenced with a 3500xL Genetic Analyzer (Applied Biosystems). Obtained results were analyzed using Variant Reporter Software v1.1 (Applied Biosystems).

### In silico protein analysis

Multiple sequence alignment (MSA) of TBC and TLDc domains of TBC1D24 was proposed using the following procedure. First, homologs of the human TBC1D24 protein (GenBank id: BAH16654.1) were identified in a PSI-Blast^34^ search (E-value threshold of 0.01, 4 iterations) performed against the NCBI non-redundant protein sequence database. Next, sequences from chordates were selected and their domain organization was predicted with hmmscan^35^. As the TBC1D24 sequence is characterized by the presence of the TBC domain followed by the TLDc domain^13^ only proteins with such domain organization were retained for further analysis. Finally, selected sequences were clustered with cd-hit^36^ using a 90% sequence identity threshold. MSA was constructed with the mafft program using the L-INS-I strategy^37^. The procedure was carried out independently for TBC and TLDc domains. Proteins with “hypothetical” or “low quality” descriptions were filtered out before clustering.

The homology model of the TBC domain was prepared using the following approach. The crystal structure of the protein from *D. melanogaster* (pdb|5hjn) was selected as a template after analyzing the results of the GeneSilico Metaserver^38^. The sequence-to-structure alignment was built using the consensus alignment approach and 3D assessment^39^ based on the FFAS, HHSearch and Psi-Blast results.The multiple sequence alignment of the family was also taken into consideration. The 3D model of the protein was built with MODELLER^40^. A model quality assessment was carried out using the MolProbity webserver^41^. Due to the presence of a nearly 40-residue long insertion in the human TLDc domain compared to the template (pdb|6r82), the model was proposed using the I-TASSER server^42^ rather than by MODELLER. To predict the putative conformation of TBC-TLDc complex in *D. melanogaster*, crystal structures of TBC and TLDc domains were used (pdb|5jhn and pdb|6r82, respectively). Protein–protein docking was carried out with the Hdock server using its default parameters^43^. For the TLDc domain, amino acid conservation was calculated using the ConSurf Server^44^ and the manually curated MSA for this domain. Visualization was done with Pymol (www.pymol.org).

### Informed consent

Written informed consent was obtained from all participants.

### Consent for publication

Participants consented to publication of nonidentifiable data.

## Results

### Cochlea involvement in patients with *TBC1D24*-related ADHL

*TBC1D24* variants causative for HL were identified in four probands (4/102), which corresponds to an approx. 4%-prevalence of *TBC1D24*-related ADHL in our cohort. All affected individuals from the four *TBC1D24* families were diagnosed with bilateral, postlingual, progressive sensorineural HL. Mean HL onset was at the earliest in the second decade of life, i.e., 19.5 y.o. in Family 1, 16.7 y.o. in Family 2, 16.3 y.o. in Family 3 and 35 y.o. in Family 4. In all families hearing thresholds had a similar down-sloping pattern and mainly mid and high frequencies were affected. Most HL patients routinely use HAs (Table 1).

**Table 1:** Results of objective audiological, neurotological and imaging examinations of probands with *TBC1D24*-related ADHL.

	Family 1 Patient IV.1 (M, 29 y.o.)	Family 2 Patient IV.12 (F, 39 y.o.)	Family 3 Patient IV.7 (F, 24 y.o.)	Family 4 Patient IV.6 (F, 39 y.o.)
Age at HL onset	18 y.o	15 y.o	8 y.o	34 y.o
PTA	Bilateral, mild to moderate	Bilateral, moderate to severe	Bilateral, severe to profound	Bilateral, mild to moderate
TEOAE	Absent	Absent	Absent	Absent
Stapedial muscle reflex	Increased	Increased	Absent	Increased
ABR	Normal	Normal	Absent	Normal
cVEMP	Normal	Normal	Normal	Normal
oVEMP	Normal	Normal	Normal	Normal
Tinnitus/vertigo	Chronic tinnitus, sporadic vertigo	Chronic tinnitus, no vertigo	Sporadic tinnitus, no vertigo	Chronic tinnitus, no vertigo
HAs or CI usage	Binaural HAs	Binaural HAs	Unilateral CI	Binaural HAs
Temporal bone CT	Normal	Normal	Normal	Normal

*ABR* auditory brainstem responses, *CI* cochlear implant, *CT* computed tomography, *cVEMP* cervical vestibular evoked myogenic potentials, *F* female, *HAs* hearing aids, *M* male, *oVEMP *ocular vestibular evoked myogenic potentials, *PTA* pure tone audiometry, *TEOAE* transient evoked otoacoustic emissions, *y.o.* years old.

**Figure 2:**
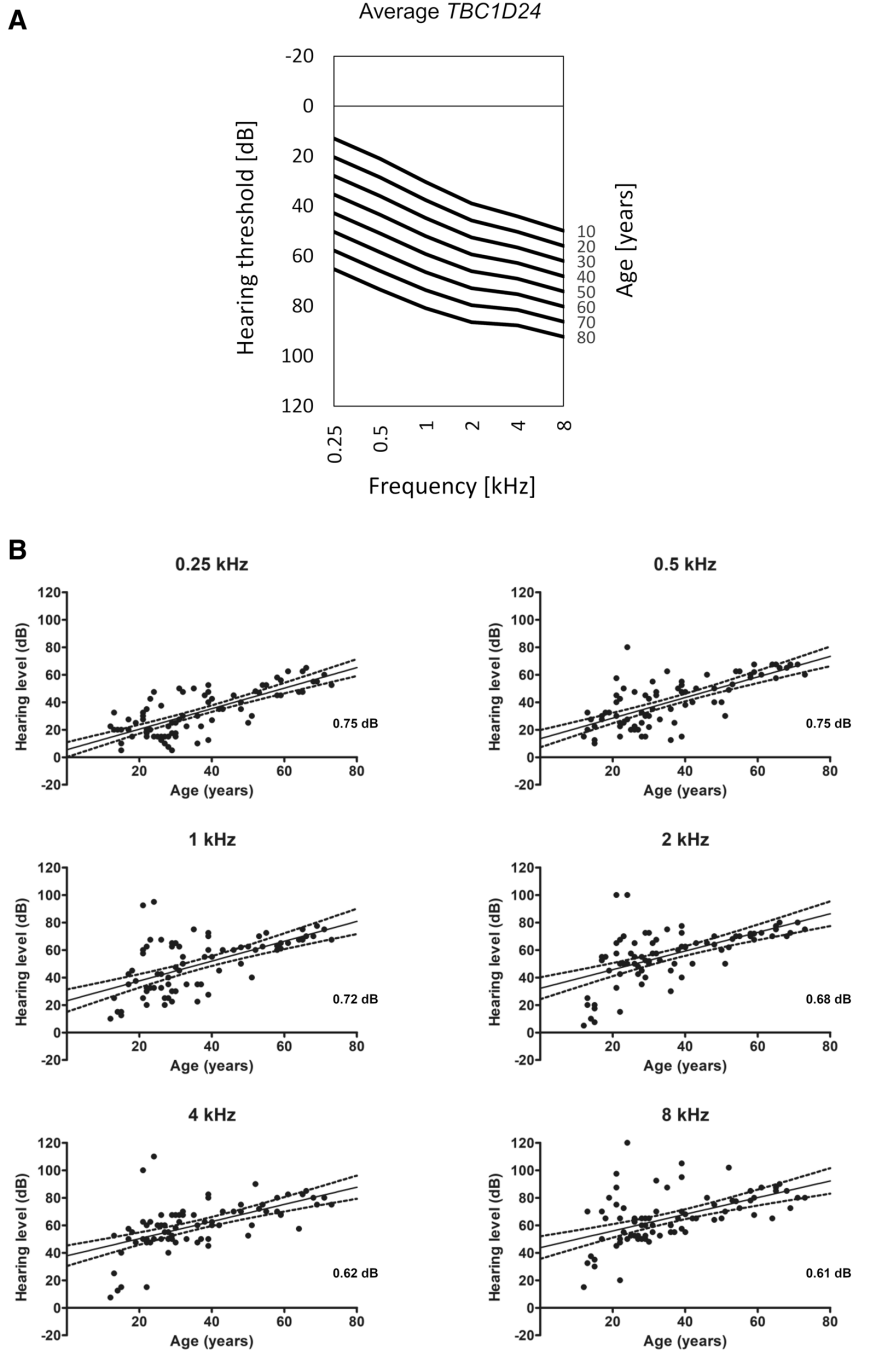
Audiological characteristics of *TBC1D24*-related ADHL. (**A**) ARTA for patients with *TBC1D24* pathogenic variants. (**B**) Rate of HL deterioration in PTA.

As shown by average ARTA (Fig. 2A) *TBC1D24*-related HL starts as mild at low and mid frequencies and moderate at high frequencies. It progresses with age (up to 80 y.o.) and becomes moderate to severe at low and mid frequencies and profound at high frequencies. The ATD in patients with *TBC1D24* pathogenic variants is statistically significant at all frequencies (p < 0.001) and ranges from 0.61 (8 kHz) to 0.75 (0.25 and 0.5 kHz) dB/year (Fig. 2B).

**Figure 3:**
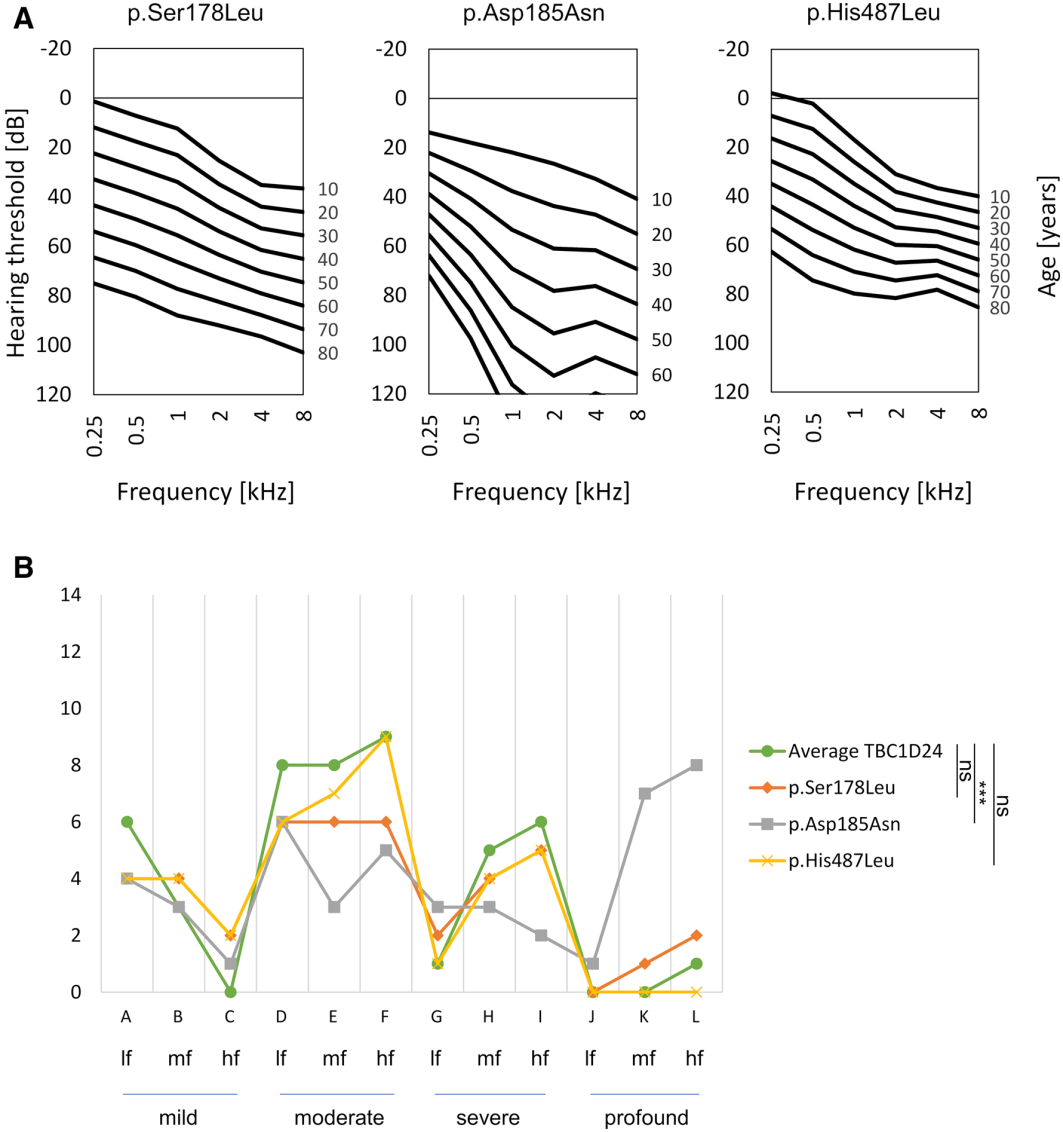
Audiological characteristics of HL observed in patients with a particular *TBC1D24* pathogenic variant. (**A**) ARTA for patients with p.Ser178Leu, p.Asp185Asn and p.His487Leu pathogenic variants. (**B**) Threshold feature array for HL pattern observed for a particular *TBC1D24* pathogenic variant. The graph represents the distribution of ARTA data points counted for different classes of HL degree and frequency (prepared based on the model proposed by Huygen et al. 2003)^19^. *lf* low frequencies, *mf* mid frequencies, *hf* high frequencies.

Analysis of PTA data of patients with the *TBC1D24* p.Ser178Leu, p.Asp185Asn and p.His487Leu variants revealed variant-dependent differences in the HL degree. In patients with p.Ser178Leu and p.His487Leu, ARTA presents mild HL at the age of 20. Later in life, HL becomes moderate to severe (Fig. 3A). The ATD is significant at all analyzed frequencies (p < 0.001) and ranges from 0.88 (4 kHz) to 1.08 (1 kHz) dB/year for p.Ser178Leu and from 0.59 (4 kHz) to 1.03 (0.5 kHz) dB/year for p.His487Leu (Supplementary Fig. S2). In patients with the p.Asp185Asn variant, ARTA shows a more severe HL over the analyzed time interval. From the age of 50 HL becomes profound at mid and high frequencies (Fig. 3A). For the *TBC1D24* p.Asp185Asn pathogenic variant, ATD is statistically significant at all frequencies (p < 0.001) and ranges from 0.83 (0.25 kHz) to 1.72 (2 kHz) dB/year (Supplementary Fig. S2). The observations were confirmed by comparing the threshold feature arrays, which revealed a statistically significant difference between HL pattern found in p.Asp185Asn patients compared to an average *TBC1D24* ARTA (Fig. 3B).

Results of additional audiological and neurotological examinations revealed cochlear involvement in the *TBC1D24*-related ADHL. No TEOAEs were recorded in the tested probands, and no or increased thresholds of stapedial muscle reflexes were observed. The vestibulocochlear nerve function measured by ABR was in line with the PTA results. No vestibular dysfunction or anatomical abnormalities of the cochleovestibular system were found. All probands reported sporadic or chronic tinnitus, one patient had sporadic vertigo (Table 1).

### Identification of *TBC1D24* pathogenic variants

**Figure 4:**
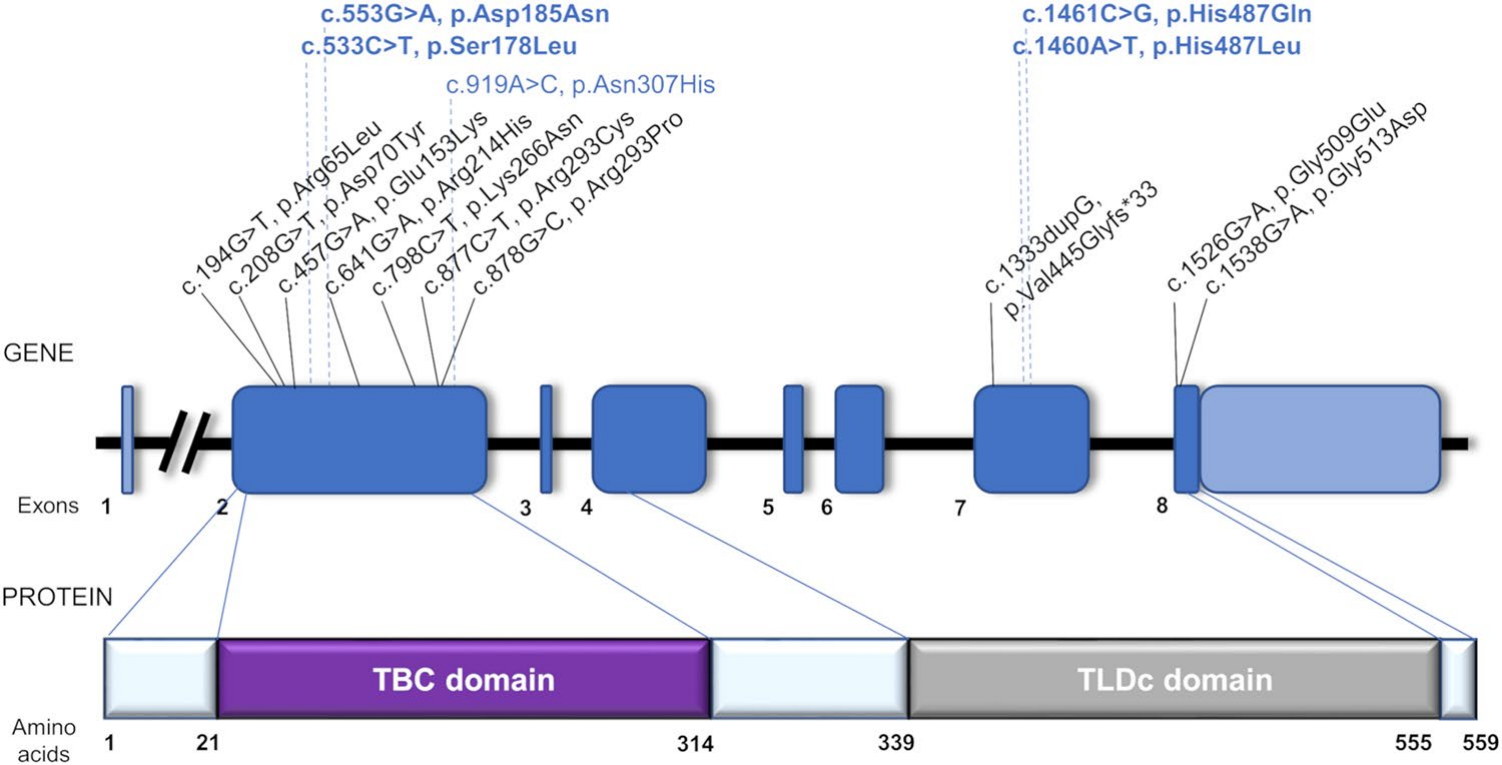
Schematic representation of *TBC1D24* gene and protein organization. Gene and protein structure are depicted based on the canonical transcript NM_001199107.1 and reference protein sequence NP_001186036.1. Previously reported *TBC1D24* pathogenic variants involved in the development of nonsyndromic ARHL (DFNB86) are written in black. Variants causative for ADHL (DFNA65) are shown in blue; variants identified in this study are bolded. Domain boundaries (TBC residues 21–314; TLDc residues 339–555) were determined based on the sequence to structure alignment between human TBC1D24 protein and Skywalker/TBC1D24 protein from *D. melanogaster*.

After performing next-generation sequencing (NGS) in four tested families very rare heterozygous variants of the *TBC1D24* gene were selected. In Families 1 and 2 were identified, respectively, a heterozygous c.533C>T, p.Ser178Leu variant and a heterozygous c.553G>A, p.Asp185Asn variant, both located in exon 2 of the *TBC1D24* gene. In Families 3 and 4, a heterozygous c.1460A>T, p.His487Leu variant and a heterozygous c.1461C>G, p.His487Gln variant were found in *TBC1D24* exon 7 (Fig. 4, Supplementary Fig. S3). All selected variants were either very rare or not present in population databases. Most computational algorithms predicted their damaging role (Table 2). No impact of the analyzed variants on *TBC1D24* RNA splicing was predicted. Family studies confirmed variants segregation with HL (Fig. 1A–D). Based on the applicable standards and guidelines, detected *TBC1D24* variants were classified as likely pathogenic^32,33^. All variants have been submitted to the Global Variome shared LOVD. No other pathogenic or likely pathogenic variants related to isolated or syndromic hereditary HL were found.

**Table 2:** Characteristics of *TBC1D24* variants detected in this study.

Family	Variant cDNA Level	Variant Protein Level	Exon	Reference SNP ID	Population frequencies			Pathogenicity predictions						
					gnomAD	UK10K	EVS	SIFT	PolyPhen-2	Mutation Taster	LRT	CADD	Revel	ACMG Classification *
Family 1	c.533C>T	p.Ser178Leu	2	rs483352866	0000008	0	0	T (0.107)	PD (0.947)	D (1)	D (9.99e−7)	D (24.2)	0.667	LP (PM2, PP1_Strong)
Family 2	c.553G>A	p.Asp185Asn	2	N/A	0	0	0	T (0.103)	PD (1.000)	D (0.99)	D (0)	D (25.5)	0.242	LP (PM2, PP1_Strong)
Family 3	c.1461C>G	p.His487Gln	7	N/A	0	0	0	D (0.024)	PD ( 0.934)	D (1)	D (0)	D (23.3)	0.303	LP (PM2, PP1_Strong)
Family 4	c.1460A>T	p.His487Leu	7	N/A	0	0	0	D (0.005)	PD (0.877)	D (1)	D (0)	D (24.4)	0.664	LP (PM2, PP1_Strong)

*ACMG classification criteria legend: *LP* likely pathogenic, *PM* moderate pathogenicity evidence, *PP_Strong* strong pathogenicity evidence, *D* damaging, *N/A* no data available, *PD* probably damaging, *T* tolerated.

The c.533C>T variant identified in Family 1 has already been described in two ADHL families^1,11^. It was the first, and for a long time, the only known *TBC1D24* variant causally involved in the development of ADHL. The second identified variant (c.553G>A) is located seven amino acids downstream of this known variant; both are placed in the TBC domain of the TBC1D24 protein. Variants identified in Families 3 and 4 are nucleotide substitutions in adjacent positions within the same codon of the *TBC1D24* sequence, resulting in an amino acid change from histidine respectively to glutamine or leucine. The latter variants are the first ADHL-related genetic alterations located in the TLDc domain of TBC1D24.

### Modeling the functional role of *TBC1D24* variants

**Figure 5:**
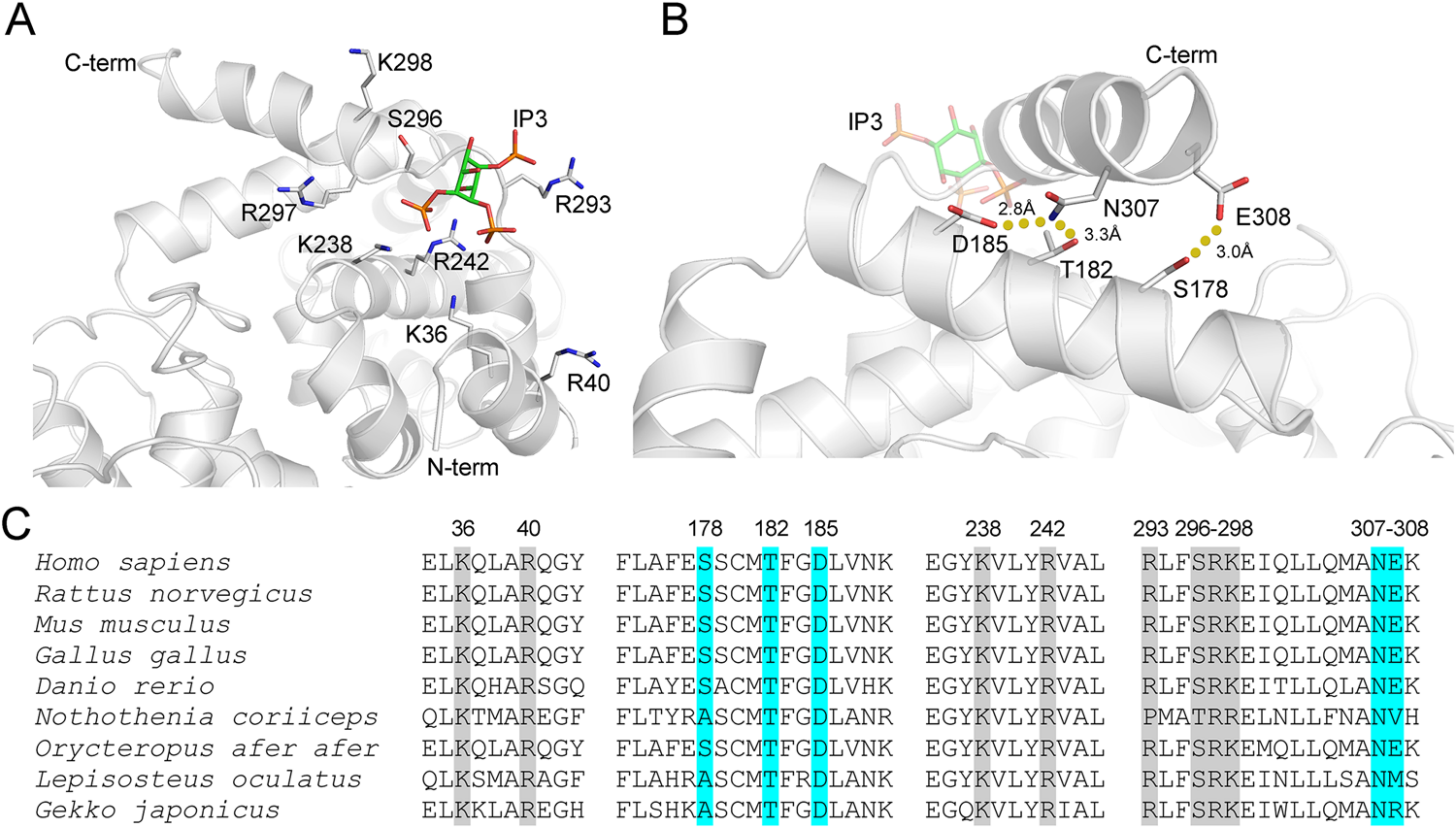
Structure of the human TBC domain from TBC1D24. (**A**) The homology model of TBC1D24 protein was superimposed over the IP_3_-Skywalker complex to obtain the structure of the human protein—IP_3_ complex. The binding sites constitute of several, conserved, mostly positively charged amino acids. (**B**) The C-terminal helix is stabilized by polar interactions between N307, E308 with T182, D185 and S178. The latter two amino acids are changed to N and L, respectively, in patients with ADHL (this study). (**C**) Multiple sequence alignment of the human TBC domain from TBC1D24 and its homologs from selected organisms. Only regions comprising the binding site residues and two helices harboring S178, T182, D185, N307 and E308 are shown. D185 is conserved among Chordata, while S178 is present only in a subset of organisms, suggesting its auxiliary role in stabilizing the C-terminal helix. Residues highlighted in grey are involved in recognizing the membrane by the TBC domain, while residues highlighted in cyan are involved in the stabilization of conformation of the C-terminal helix of the TBC domain.

**Figure 6:**
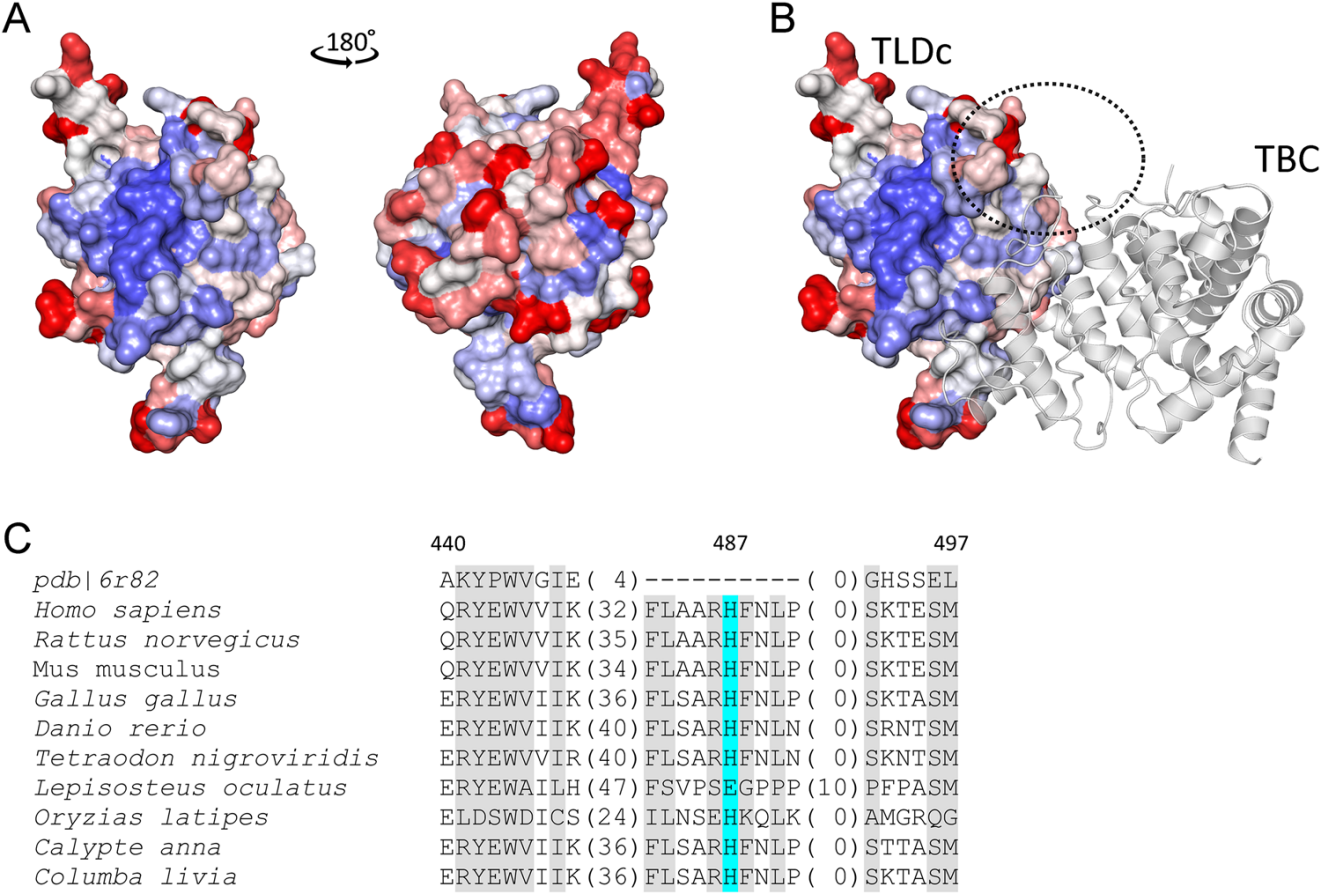
Structure of the human TLDc domain from TBC1D24. (**A**) The residues conservation for Chordata TBC1D24 mapped onto the surface of pdb|6r82. Two faces of the protein are shown. Conserved residues are colored blue, while variable ones are colored red. (**B**) The possible orientation of TBC and TLDc domains in TBC1D24 protein as proposed by HDock. The TLDc domain is colored using the same scheme as in (**A**). TBC domain is presented as cartoon. The dotted sphere represents the putative position of the loop harboring H487. (**C**) Multiple sequence alignment of the TLDc domain from TBC1D24 protein and its homologs from selected organisms. Only the loop harboring His487 is shown. Both termini (including His487 highlighted in cyan) of this loop are conserved among Chordata, while the middle of the loop shows the significant sequence and structural variability. Residues highlighted in gray are conserved across Chordata (a specific residue is present in more than 80% of analyzed sequences).

The homologs of the human TBC1D24 protein were collected and clustered using the procedure described in the “Materials and methods” section. For the TBC domain, it resulted in 55 proteins that represent the sequence variability of the TBC domain from the TBC1D24 family. Analysis of the multiple sequence alignment (MSA for selected residues and species is presented in Fig. 5C) allowed us to identify that the amino acids crucial for interactions with IP_3_ in *D. melanogaster*, i.e. Lys75, Arg79, Lys277, Arg281, Arg335, Gly336 and Thr339, are either unchanged or substituted with positively charged residues—Lys36, Arg40, Lys238, Arg242. Arg293, Arg297, Lys298 (residues are numbered according to the human sequence). This might lead to even stronger interactions between the protein and IP_3_ in humans compared to *D. melanogaster*. Additionally, the MSA analysis revealed that both residues involved in ADHL, i.e., Asp185 and Ser178, have a different conservation level. In only one case Asp185 is replaced with glutamic acid, preserving the negatively charged residue. However, Ser178 is present only in 25 out of 55 sequences and is often replaced by other small residues like alanine or threonine. A similar analysis was independently performed for the TLDc domain, resulting in 118 protein sequences. According to the MSA, His487 is a part of a long loop (between β5 and β6) with variable length across different Chordata (MSA for selected residues and species is presented in Fig. 6C). In the TBC1D24 homolog from *D. melanogaster,* this loop is 18 residues long (from Pro511 to Phe528), while, in humans, it spans 56 residues (from Glu443 to Phe498). Despite high sequence variability observed in the middle of this loop, its termini are conserved across Chordata. His487 is localized near the C-terminus of this loop and is observed in 81 sequences. Since the MSA reflects the sequence variability of TLDc domains, the remaining 37 sequences that lacked H487 were verified manually. It turned out that these sequences originated from 36 organisms. The majority of them were either a partially solved sequence or represented one of many isoforms of the TLDc domain present in that species. Only for ten species we could not find an isoform of TBC1D24 with His at position 487. It can be therefore concluded that His487 is a conserved residue among Chordata.

To analyze the putative role of p.Asp185Asn and p.Ser178Leu mutations, a homology model of the human TBC domain from TBC1D24 was constructed. Asp185 is located outside the active site on the 6th helix and does not interact directly with IP_3_ (Fig. 5A). The distance to the closest Cα atom of a residue from the binding site (Lys238) is nearly 14 Å. According to our model Asp185 forms a hydrogen bond with Asn307 (Fig. 5B) located in the C-terminal helix of the TBC domain. Asn307, like Asp185, is also conserved among Chordata (with only two exceptions where histidine is present). The C-terminal helix itself is a part of the IP_3_ binding site, as its N-terminal fragment harbors Ser296, Arg297, and Lys298. Thus, it is plausible that the Asp185-Asn307 bond is a part of a network of interactions responsible for maintaining a proper geometry of the 6th and C-terminal helices, which influences the position of Arg297 and Lys298 within the binding site. Close inspection of our model revealed that two additional polar interactions are part of this network. First, Asn307 forms an additional hydrogen bond with Thr182 residue, also conserved among Chordata. The second interaction in this model involves Ser178 and Glu308 (Fig. 5B). However, as both Ser178 and Glu308 are present only in a subset of chordates’ TBC1D24, this hydrogen bond may perform only an auxiliary role. Intriguingly, p.Ser178Leu was already linked with ADHL^1,11^ and based on the model proposed by Parzefall et al., Ser178 interacts with Asn307^12^. Considering the close proximity of both Asn307 and Glu308 residues, Ser178 interaction with either of these two residues is equally probable.

As mentioned above, His487 is part of a long flexible loop that hinders the probability of correctly identifying its position in relation to the rest of the TLDc domain. I-Tasser, employed to predict the possible orientations of that fragment, produces several, equally likely and quite diverse conformations. However, in most cases, residue conservation indicates amino acid importance in maintaining the proper 3D structure of a protein, its function or ability to bind to other protein. We investigated the possible role of His487 in the binding of the TBC domain. This hypothesis was put forward as we observed that the putative position of the loop harboring His487 is close to a patch of several conserved, solvent-exposed residues (Fig. 6A). To obtain a putative conformation of the TBC-TLDc complex, we performed a protein–protein docking using available crystal structures of these domains from *D. melanogaster*. Analysis of the top ten conformations revealed that the TLDc domain interface involves residues from this conserved patch (Fig. 6B). No such consistency was observed for the TBC domain. Considering the His487 proximity to this region, p.His487Gln and p.His487Leu might be responsible for weakening the interface between both domains of the TBC1D24 protein.

## Discussion

Since the first association of *TBC1D24* with isolated HL in 2014, the number of its identified variants causing a recessive form of HL has been steadily growing, currently reaching as many as ten. The case has been different for *TBC1D24* variants involved in ADHL development. The first ADHL-related *TBC1D24* pathogenic variant (p.Ser178Leu) was also identified in 2014, found in parallel in a European and a Chinese family^1,11^. Before 2020, no other *TBC1D24* alteration has been associated with this condition. The second *TBC1D24*-related ADHL variant (p.Asn307His) has been reported recently in a study of two unrelated HL families from Austria and the UK^12^ (Table 3). Altogether, the data suggested that the involvement of *TBC1D24* in ADHL might be considered relatively rare and exceptional.

**Table 3:** Comparison of demographic, molecular, and clinical findings in patients with isolated HL due to *TBC1D24* pathogenic variants.

Family # and origin		Consanguinity	Tested patients with HL (No.)	Type of HL, onset	Reference SNP ID	Variant cDNA level	Variant Protein level	Protein domain	Ref.
1	Pakistani	Y	11	Profound, congenital	rs587777147	c.208G>T	p.Asp70Tyr	TBC	^6^
					rs587777147	c.208G>T	p.Asp70Tyr		
2	Pakistani	Y	9	Profound, congenital	rs587777147	c.208G>T	p.Asp70Tyr	TBC	
					rs587777147	c.208G>T	p.Asp70Tyr		
3	Pakistani	Y	4	Profound, congenital	rs587777147	c.208G>T	p.Asp70Tyr	TBC	
					rs587777147	c.208G>T	p.Asp70Tyr		
4	Pakistani	Y	7	Profound, congenital	rs199700840	c.878G>C	p.Arg293Pro	other	
					rs199700840	c.878G>C	p.Arg293Pro		
5	Moroccan	Y	3	Severe to profound congenital	rs200324356	c.641G>A	p.Arg214His	TBC	^7^
					N/A	c.1333dupG	p.Val445Glyfs*33	TLDc	
6	Moroccan	Y	2	Severe to profound congenital	rs376712059	c.457G>A	p.Glu153Lys	TBC	
					N/A	c.798G>T	p.Lys266Asn	TBC	
7	Israeli	Y	3	Profound, congenital	rs878853232	c.194G>T	p.Arg65Leu	TBC	^8^
					rs878853232	c.194G>T	p.Arg65Leu	TBC	
8	Czech	N	1	Profound, congenital	N/A	c.1526G>A	p.Gly509Glu	TLDc	^9^
					N/A	c.1538G>A	p.Gly513Asp	TLDc	
9	Chinese	N	1	Profound, congenital	rs367966267	c.877C>T	p.Arg293Cys	Other	^10^
					rs367966267	c.877C>T	p.Arg293Cys		
10	USA	N	10	Progressive, 3rd decade	rs483352866	c.533C>T	p.Ser178Leu	TBC	^1^
11	Chinese	N	9	Progressive, 3rd decade	rs483352866	c.533C>T	p.Ser178Leu	TBC	^11^
12	Austrian	N	7	Progressive, 2nd decade	N/A	c.919A>C	p.Asn307His	TBC	^12^
13	British	N	3	Progressive. 3rd decade	N/A	c.919A>C	p.Asn307His	TBC	
14	Polish	N	6	Progressive, 2nd decade	rs483352866	c.533C>T	p.Ser178Leu	TBC	Present study
15	Polish	N	12	Progressive, 2nd decade	N/A	c.553G>A	p.Asp185Asn	TBC	
16	Polish	N	6	Progressive, 2nd decade	N/A	c.1461C>G	p.His487Gln	TLDc	
17	Polish	N	5	Progressive, 3rd decade	N/A	c.1460A>T	p.His487Leu	TLDc	

*HL* hearing loss, *N* no, *N/A* no data available, *Y* yes.

Our study shows that the role of *TBC1D24* in ADHL may be underestimated. After testing 102 multigenerational families fulfilling strict criteria for the autosomal dominant inheritance of HL, the pathogenic *TBC1D24* variant was detected in almost 4% (4/102). The involvement of *TBC1D24* in ADHL can be even more significant than that. There are two additional ADHL families in our cohort whose probable underlying cause of HL is a defective *TBC1D24* (data not shown). As further investigations are required to unequivocally confirm the *TBC1D24* pathogenic potential, the families have not been presented here. We can also speculate that there were other patients with *TBC1D24*-related ADHL who have been excluded from our analysis because (i) their pattern of HL inheritance was mimicking a mitochondrial mode of HL inheritance, or (ii) the variants arose de novo*,* and the family history of HL was negative.

It is important to note that the contribution of one particular gene to ADHL development does not exceed a dozen percent. Among the currently known 50 genes implicated in ADHL development (https://hereditaryhearingloss.org/dominant-genes; accessed 03/2021), there is no single major gene responsible for ADHL. It varies among populations, but pathogenic variants in *TECTA, KCNQ4*, *WFS1,* or *MYO6* are more frequent than other ADHL genes^45,46^. Pathogenic variants in *MYO6*, *KCNQ4*, *WFS1*, *GSDME* (DFNA5) or *TECTA* were also repeatedly found in our ADHL cohort (unpublished data). While *MYO6* pathogenic variants explained 11% of HL causes in the studied ADHL families (data not shown), the remaining genes did not exceed 4%, as observed for *TBC1D24*.

The *TBC1D24* gene (OMIM *613577) has five transcripts (www.ensembl.org; accessed 02/2021) encoding different isoforms found in multiple human tissues. The highest expression of the *TBC1D24* gene was observed in the brain, but it was also found in testis, skeletal muscle, heart, kidneys, lung, and liver^47^. In the human auditory system, *TBC1D24* mRNA and protein were detected in hair cells and spiral ganglion neurons^48^. Clinically, our patients presented typical features of isolated sensorineural HL with cochlea involvement, impaired hair cell function, normal function of the auditory nerve and normal anatomical ear structures. Their HL was progressing slowly and was accompanied by tinnitus. Some differences in the HL progression rate were mutation-specific and the highest progression rate was observed for p.Asp185Asn. It was significantly higher than the average *TBC1D24* progression rate calculated based on all available data.

The majority of our patients benefit from using hearing aids, even throughout their lifetime. An exception in this regard was the proband from Family 3. Unlike other patients with *TBC1D24*-related ADHL, including her family members, she was diagnosed with HL at 8 and received a cochlear implant at 22. In this patient we could not identify any additional genetic or environmental factors that could aggravate her HL. Generally, the age of HL onset in our patients ranged between the second and the fourth decade of life, which was in line with the data reported previously^1,11,12^.

All of the ADHL-related *TBC1D24* pathogenic variants, identified so far represent missense changes that based on bioinformatic predictions, do not affect *TBC1D24* transcript formation and are scattered throughout the gene. It is in line with the observation that the type and location of the *TBC1D24* variant cannot predict the associated phenotype^49^. Out of three novel variants identified here, the p.Asp185Asn localizes in the N-terminal protein region seven amino acids away from p.Ser178Leu, both within the TBC domain. The other two alterations (p.His487Gln and p.His487Leu) localize in the more C-terminal part of the protein corresponding to the TLDc domain. Interestingly, a frameshift variant involving this codon (p.His487Glnfs*71) was found in a patient with the DOORS syndrome^50^.

Both Ser178 and Asp185 appear to serve a similar structural role in establishing the proper conformation of the TBC1D24 C-terminal helix and, consequently, the IP_3_ binding site. Considering their structural proximity, both mutations identified in this work, i.e., p.Ser178Leu and p.Asp185Asn should have a common molecular mechanism leading to HL. In our opinion, both mutations would most likely influence the conformation of the C-terminal helix. That would affect the orientation of binding site residues, i.e., Arg293, Arg297 and Lys298, which could result in the disruption of some of the interactions between IP_3_ and the TBC1D24 protein. Consequently, the decreased binding affinity would be responsible for impaired vesicular traffic that could lead to HL. The mutations affecting the TLDc domain, target conserved His487 amino acid. This residue is located in the long loop near the putative interface between TBC and TLDc domains. Thus, these mutations can destabilize the formation of this complex.

In summary, our data revealed that *TBC1D24* should be considered an important contributor to ADHL. As ADHL shows similarities with presbyacusis, *TBC1D24* can be proposed as a candidate gene for the age-related HL. Similar to other *TBC1D24*-associated disorders, variants causative for ADHL can be found in different parts of the gene, which makes the studies of its functional role even more challenging. Based on the in silico analysis of homology models, we assume that the positions of amino acids involved in ADHL development are responsible for maintaining a proper geometry of the TBC1D24 protein, which is crucial for its interactions with phosphoinositides.

## Supplementary Information

Supplementary Figure S1.

Supplementary Figure S2.

Supplementary Figure S3.

## Data Availability

Results of the bioinformatic analysis can be found in a GitHub repository: https://github.com/SFGLab/TBC1D24.

## Abbreviations

ABRs: Auditory brainstem responses
ARTA: Age-related typical audiogram
ATD: Annual threshold deterioration
ADHL: Autosomal dominant hearing loss
OAE: Otoacoustic emissions
nHL: Normal hearing level
cVEMP: Cervical vestibular evoked myogenic potentials
oVEMP: Ocular vestibular evoked myogenic potentials
