# Supplementary figures and images for: TBC1D24 emerges as an important contributor to progressive postlingual dominant hearing loss
Source: Sci Rep. 2021 May 13;11:10300. doi: 10.1038/s41598-021-89645-y (PMC8119487; doi:10.1038/s41598-021-89645-y)

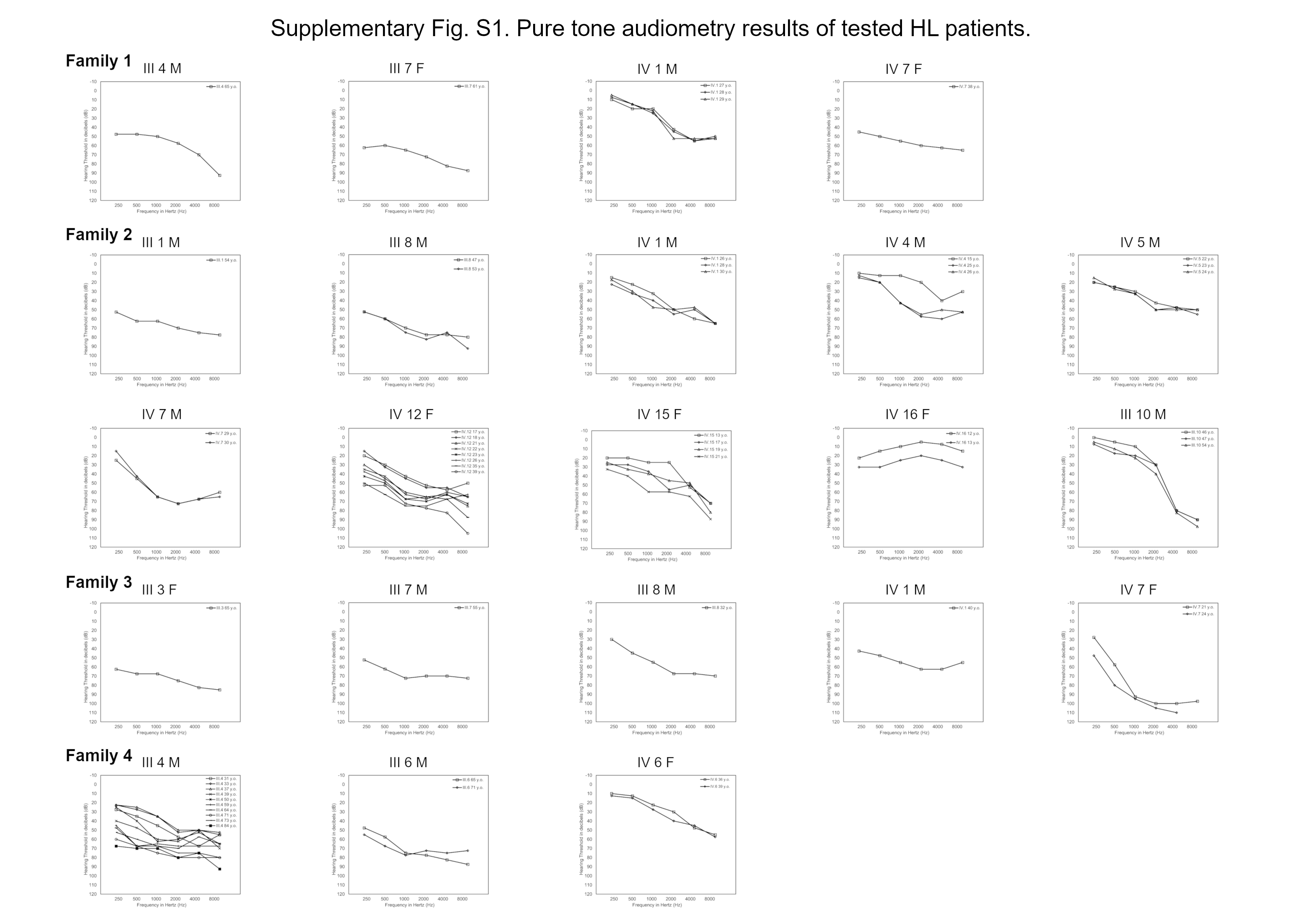

Supplement: Supplementary file 1 — Supplementary Figure S1. [file 41598_2021_89645_MOESM1_ESM.tif]

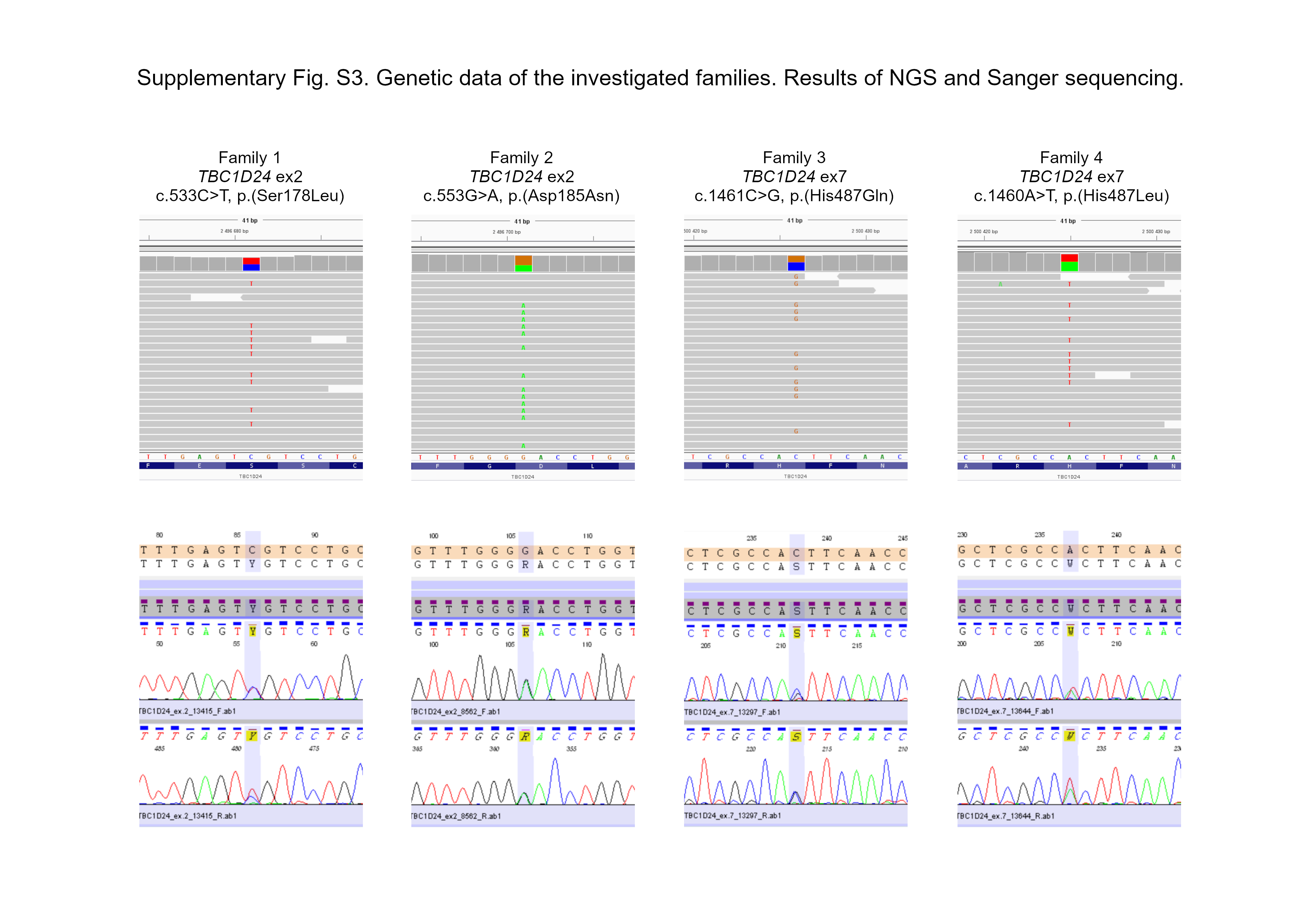

Supplement: Supplementary file 3 — Supplementary Figure S3. [file 41598_2021_89645_MOESM3_ESM.tif]
